# A systematic review and meta-analysis of cardiovascular diseases and associated factors among diabetes mellitus patients in Ethiopia

**DOI:** 10.1186/s12872-023-03443-0

**Published:** 2023-08-21

**Authors:** Tadele Lankrew Ayalew, Kirubel Eshetu Haile, Mulualem Gete Feleke, Bitew Tefera Zewudie, Tesfaye Yitna Chichiabellu

**Affiliations:** 1 Department of Nursing, School of Nursing, College of Health Sciences and Medicine, Wolaita Sodo University, P.O. Box 138, Sodo, Ethiopia; 2 Department of Nursing, College of Health Science and Medicine, Wolkite University, Wolkite, Ethiopia

**Keywords:** Cardiovascular disease, Diabetes mellitus, Ethiopia

## Abstract

**Background:**

Cardiovascular disease (CVD) is the most prevalent complication and the leading cause of death and disability among patients with diabetes mellitus (DM). Over time, diabetes-related cardiovascular disease has become more common worldwide. The aim of this study was to determine the cumulative prevalence of cardiovascular disease and associated factors among diabetic patients in Ethiopia.

**Objective:**

The main aim of this review was to estimate the pooled prevalence of cardiovascular disease and its associated factors among diabetic patients in Ethiopia.

**Methods and materials:**

This review was searched using PubMed, Google, and Google Scholar search engines, and was accessed using medical subject heading (MeSH) terms for studies based in Ethiopia. Excel was used to extract the data. With a random-effects model, STATA Version 14 was used for all statistical analyses. The studies' heterogeneity and funnel plot were both examined. The study domain and authors' names were used in the subgroup analysis.

**Results:**

In this systematic review, 12 studies totaling 2,953 participants were included. The estimated overall prevalence of cardiovascular disease among diabetic patients in Ethiopia was 37.26% (95% CI: 21.05, 53.47, I^2^ = 99.3%, *P* ≤ 0.001). Study participants’ age older than 60 years (AOR = 4.74, 95%CI: 1.05, 8.43), BMI > 24.9kg/m^2^ (AOR = 4.12, 95% CI: 2.33, 5.92), triglyceride > 200mg/dl (AOR = 3.05, 95% CI: 1.26, 4.83), Hypertension (AOR = 3.26, 95% CI: 1.09, 5.43) and duration of DM > 4 years (AOR = 5.49, 95% CI: 3.27, 7.70) were significantly associated with cardiovascular disease.

**Conclusions:**

In conclusion, diabetic patients face a serious public health risk from cardiovascular disease. This review found the following factors, which is independent predictors of cardiovascular disease in diabetic patients: age over 60, BMI > 24.9kg/m^2^, triglycerides > 200 mg/dl, hypertension, and diabetes duration > 4 years. The results emphasize the need for a prospective study design with a longer follow-up period to assess the long-term effects of CVD predictors in diabetic patients as well as the significance of paying attention to cardiovascular disease in diabetic patients with comorbidity.

**Supplementary Information:**

The online version contains supplementary material available at 10.1186/s12872-023-03443-0.

## Introduction

Non-communicable diseases (NCDs) have surpassed infectious diseases as the leading cause of death worldwide, accounting for about 36 million fatalities annually [1]. Cardiovascular disease (CVD) is the main danger to human health and development, followed by cancer, persistent respiratory conditions, and diabetes. About 35 million people per year die from it, with poorer nations accounting for 85% of those deaths [2]. The word "CVDs" refers to a set of diseases and injuries that have an impact on the heart and the tissues that support it. Deep vein thrombosis (DVT), stroke, angina, congestive heart failure, coronary heart disease, and peripheral arterial disease are only a few of the conditions referred to as "CVDs" (but they are not the only ones) [2, 3]. The main cause of early death and disability in diabetic people is CVDs [4]. This has a substantial effect on the rising expense of healthcare. According to studies, the proportion of people who die from CVDs before their time ranges from 42% in low-income countries to 4% in high-income countries, showing the growing disparities between the populations living in different countries [4–6].

Obesity, poor diet, high blood pressure, and diabetes mellitus are common, and their prevalence is rising more quickly than their burden can be alleviated. Despite the fact that CVD is preventable, it is responsible for around 31% of all fatalities globally, and more than 3 million of those deaths occurred before the age of 60 year [2, 7–9]. In low- and middle-income nations, almost 80% of fatalities from CVD-related causes occurred. According to the Global Disease Burden Report 2015, population growth and aging have led to a rise in the percentage of CVD-related mortality in many of the world's poorer regions. In Eastern and Central Sub-Saharan Africa, the disease is more common than in Western and Southern Sub-Saharan Africa [2, 7, 10, 11].

Excessive alcohol use, energy-dense diets, hypercholesterolemia, diabetes, sedentary lifestyles, overweight/obesity, age, sex, family history, and ethnicity are only a few of the numerous and diverse causes of CVD [2, 12, 13]. Due to the necessity for information on persons who do not attend the medical institution, it is notoriously difficult to estimate the prevalence of CVD in a population. It is challenging to quantify the global prevalence of CVD due to the fact that different nations report prevalence that was evaluated using various approaches. Estimating the CVD burden is very challenging in sub-Saharan African countries like Ethiopia [2, 14, 15].

The World Health Organization estimates that non-communicable diseases caused 30% of deaths in Ethiopia in 2014, with cardiovascular disease being responsible for 9% of these fatalities [2, 11]. Diabetes-related premature mortality increased by 5% between 2000 and 2016. Between 2000 and 2019, the age-standardized death rates from diabetes rose by 3%. Notably, cardiovascular (CV) events are a leading factor in the increased risk of early death in patients with type 2 diabetes mellitus (T2DM) and are a growing concern to human health globally [16, 17].

The major cause of death for DM patients is cardiovascular disease (CVD), which can shorten life expectancy by up to ten years. It should be noted that type 2 diabetes mellitus (T2DM) makes up about 90% of all cases of diabetes. Additionally, DM patients' CVD is more severe than that of non-DM individuals. Over a 7-year period, the mortality rates from cardiovascular causes were 15.4% for DM patients without a history of myocardial infarction (MI) and 42.0% for those with a history of MI. Cardiovascular death rates were 2.1% for those without type 2 diabetes and 15.9% for those who had it [4, 16, 18].

The main cardiovascular disorders (CVDs) linked to diabetes mellitus (DM) include peripheral arterial disease, coronary artery disease (CAD), and ischemic heart disease, heart failure, and stroke. These consequences could result in the death of at least 50% of DM patients [4, 16, 19].

The most effective way to prevent cardiovascular problems in diabetes individuals appears to be multifactorial risk factor reduction (glycemic management, smoking cessation, diet, exercise, rigorous blood pressure control, therapy of dyslipidemia) [20, 21]. As a result, recommendations for managing and preventing the start of CVD have been added to diabetes treatment guidelines. Regulatory agencies place an increasing emphasis on the need for anti-diabetic medications to demonstrate cardiovascular safety and benefits, particularly for significant cardiovascular events such cardiovascular mortality, non-fatal MI, and stroke. Numerous cardiovascular outcomes studies (CVOT) have been done in accordance with these regulatory requirements, proving the link between a specific anti-diabetic drug and a reduced risk of CVD [22, 23].

We need up-to-date CVD prevalence rates among DM patients due to the rising emphasis on properly treating individuals with both CVD and DM. Healthcare practitioners, healthcare policy makers, and health economic analysts will need this information in particular to make decisions at the clinical and policy levels.

However, there hasn't been a current national assessment in Ethiopia on the prevalence of CVD and related variables among diabetics. Therefore, the aim of this systematic literature review was to assess the prevalence of CVD and identify the related factors among diabetic patients in Ethiopia.

## Methods

### Objectives

The primary objective of this review was to assess the pooled prevalence of CVD among DM patients. The secondary objective was identifying the associated factors and investigating any regional differences in Ethiopia.

### Study design and search strategy

We searched studies of cardiovascular diseases and associated factors among diabetic patients in Ethiopia by using Google Scholar, Pub Med, Cochran Library, and Ethiopian University Repository online. We check the database at (http://www.library.ucsf.edu) and the Cochrane library to ensure this had not been done before and to avoid duplication. The reference lists already identified were screened to retrieve articles. Articles were searched using MESH terms on the cardiovascular diseases and associated factors among diabetic patients in Ethiopia. We strictly follow Preferred Reporting Items for Systematic and Meta-Analysis (PRISMA) protocols to estimate the cardiovascular diseases and associated factors among diabetic patients in Ethiopia.

### Criteria for eligibility

Inclusion criteria: We agree on the following inclusion standards for this meta-analysis and systematic review in order to include articles in this review.

1. Participants: Including those who reside in Ethiopia.
2. Community or institutional-based research carried out in Ethiopia;
3. Study design: All observational study designs, including cross-sectional, case–control, and cohort studies, were included.
4. Setting: Only Ethiopia was the location of the studies.
5. Study: Up until the final date of data analysis, all studies—both published and unpublished—that were published in the form of journal articles, master's theses, and dissertations were included.
6. Language: This study only took into account the English language.

Exclusion criteria: omitted papers that required two or more emails between the authors' principals to completely make accessible.

### Operational definition

Cardiovascular disease (CVD): is a medical term that refers to diseases of the heart and blood vessels. It encompasses all conditions that affect the heart and circulatory system of the body, including coronary heart disease (angina and heart attacks), hypertension (high blood pressure), stroke, and peripheral vascular disease (PVD), which is any condition that affects the circulatory system that is not related to the brain or the heart [24].

Hypertension: is defined as a blood pressure having a systolic blood pressure (SBP) ≥ 140 mm Hg or diastolic blood pressure (DPB) ≥ 90 mm Hg on at least two measurement occasions [25].

Regular physical exercise: Performing physical activity three days/week for 20–30 min duration [26].

Body mass index (BMI): is a measure of weight adjusted for height, calculated as weight in kilograms divided by the square of height in meters (kg/m^2^). It classified as normal if BMI is between 18.5 and 24.9 kg/m^2^ (18.5–24.9 kg/m^2^) and above normal if BMI is above 24.9 kg/m^2^ [27].

### Quality assessment and data collection

Joanna Brings Institute Meta-Analysis of Statistics Assessment and Review Instrument (JBI-MAStARI) was used for critical appraisal of the study.

Joanna retrieved studies and was assessed for inclusion using their title and abstracts. Then a full review of articles for the quality of assessment was done before selecting for the final review. The details of studies that met the inclusion criteria were imported into the Joanna Briggs Institute’s System for the Unified Management, Assessment and Review of Information (JBI SUMARI, The Joanna Briggs Institute, Adelaide, Australia) critical appraisal tools to evaluate the quality of all studies [28]. All authors independently assessed the article title and abstract for inclusion in the review based on established article selection criteria, appraising the quality of the studies by criteria adapted for reporting prevalence data and cross-sectional studies. Studies were considered low risk if a score of seven and above on the quality assessment indicators (Table 1). Any discrepancy which arose between the reviewers were in the review process was solved through discussion with other reviewers.

**Table 1:** Critical appraisal results of eligible studies in the systematic review and meta-analysis on the prevalence of cardiovascular disease and associated factors among diabetic patients in Ethiopia, 2022

Authors	Q1	Q2	Q3	Q4	Q5	Q6	Q7	Q8	Q9	Total
Gebisto R. et al. [29]	Y	Y	Y	Y	Y	N	Y	Y	Y	8
Adane T., etal [30]	Y	N	Y	Y	Y	Y	Y	Y	Y	8
Tekabe A.,et al. [31]	Y	Y	Y	Y	Y	Y	Y	U	Y	8
Senbeta G., et al. [32]	Y	Y	Y	Y	Y	Y	Y	Y	Y	9
Lemma D.,et al. [33]	Y	Y	Y	Y	Y	Y	Y	Y	Y	9
Hailemaryam A., et al [34]	Y	Y	Y	Y	N	Y	Y	Y	Y	8
Yonas A., et al. [35]	Y	Y	Y	Y	Y	Y	U	Y	Y	8
Birhanu W.,et al. [36]	Y	Y	Y	Y	Y	Y	Y	U	Y	8
Solomon A.,et al. [11]	Y	N	Y	Y	Y	Y	Y	Y	Y	8
Dessie A.,et al. [12]	Y	Y	Y	Y	Y	Y	Y	Y	Y	9
Solomon A.,et al. [37]	Y	Y	Y	Y	Y	Y	Y	Y	Y	9
Daba A.,et al. [38]	Y	Y	Y	Y	Y	N	Y	Y	Y	8

### Data extraction

The data extraction was done using a tool developed by the 2014 Joanna Brings Institute Reviewers’ Manual data extraction form by three authors (TL, BT, and MG) [28]. The data extraction tool includes information on the title, author, and year of study, publication year, study design, sample size, study participants, study area, response rate, and the proportion of cardiovascular disease among diabetic patients in Ethiopia. Articles that fulfilled the predefined criteria were used as a source of data for the final analysis. The reviewers cross-checked it to ensure consistency. Any discrepancy was solved through discussion with other authors and the procedure was repeated to overcome the difference, which resulted during extracting every single study.

### Methodological quality assessment of studies

The methodological quality of included studies was appraised using a modified and predefined checklist to assess the methodological quality aspect of cardiovascular disease among diabetic patients reported.

A score of yes was given for an item if meeting the methodological criteria. A score of no was given for an item is not meeting the methodological criteria, and if an item neither met the criteria nor described the related parameter sufficiently was give unclear. Here, we use the above terms to screen the eligible articles for systematic review and meta-analysis. The point is that both ‘’No and neither yes or no’’ articles illegible for this systematic review and meta-analysis. Here the JBI-MAStARI requires for the use as a methodological tool, specifically for assessing risk of bias with nine items modified quality of life assessment checklist. According to Newcastle–Ottawa quality assessment, Scale (NOS) score seven or more for cross- sectional studies was accepted. Based this a score of 7 or more out of 9 acceptable for this review [39].

List of questions to assess the methodological quality of studies on cardiovascular diseases and associated factors among diabetic patients in Ethiopia.

- Q1 = was the sample frame appropriate to address the target population?
- Q2. Were study participants sampled appropriately.
- Q3. Was the sample size adequate?
- Q4. Were the study subjects and the setting described in detail?
- Q5. Was the data analysis conducted with sufficient coverage of the identified sample?
- Q6. Were the valid methods used for the identification of the condition?
- Q7. Was the condition measured in a standard, reliable way for all participants?
- Q8. Was there appropriate statistical analysis?
- Q9. Was the response rate adequate, and if not, was the low response rate managed appropriately?

### Outcome of interest

The first outcome of interest for this systematic review and meta-analysis was the prevalence of cardiovascular diseases among diabetic patients in Ethiopia. Moreover, the pooled prevalence of cardiovascular diseases among diabetic patients and associated factors was computed. The second outcome variable is a factor associated with the prevalence of cardiovascular diseases among diabetic patients in Ethiopia and was computed by using the log and ratio. Twelve studies were included in this review.

### Heterogeneity and publication bias

The funnel plot test, I^2^, and its accompanying *p*-value were used to determine the degree of heterogeneity. The heterogeneity test results were classified as low, medium, and high heterogeneity using values of 25%, 50%, and 75%. A random effect analysis model was utilized for results that were statistically significant for heterogeneity. In order to determine the statistical importance of publication bias, the Egger regression asymmetry test was utilized [29].

### Data processing and analysis

The data were entered using Microsoft Excel. The Meta-analysis was conducted using Stata 14 software. Forest plots were used to present the combined estimate with the 95% confidence interval (CI). The estimated pooled prevalence of health-related quality of life among cancer patients in Ethiopia was computed with 95% CI. Subgroup analysis was done by region, year of the study period, and study participants. Additionally, a univariate meta-regression model was applied by taking the sample size, publication year, and quality scores of each primary study to investigate the sources of heterogeneity. Finally, a forest plot figure was used to present the point proportions with their 95% CI of the primary studies. The heterogeneity of included studies was evaluated with I^2^ statistics. Based on I^2^ statistics, a value less than 25% were considered low heterogeneity, between 50 and 75% medium heterogeneity and greater than 75% were considered as high heterogeneity [30].

## Results

### Studies identified

**Fig. 1:**
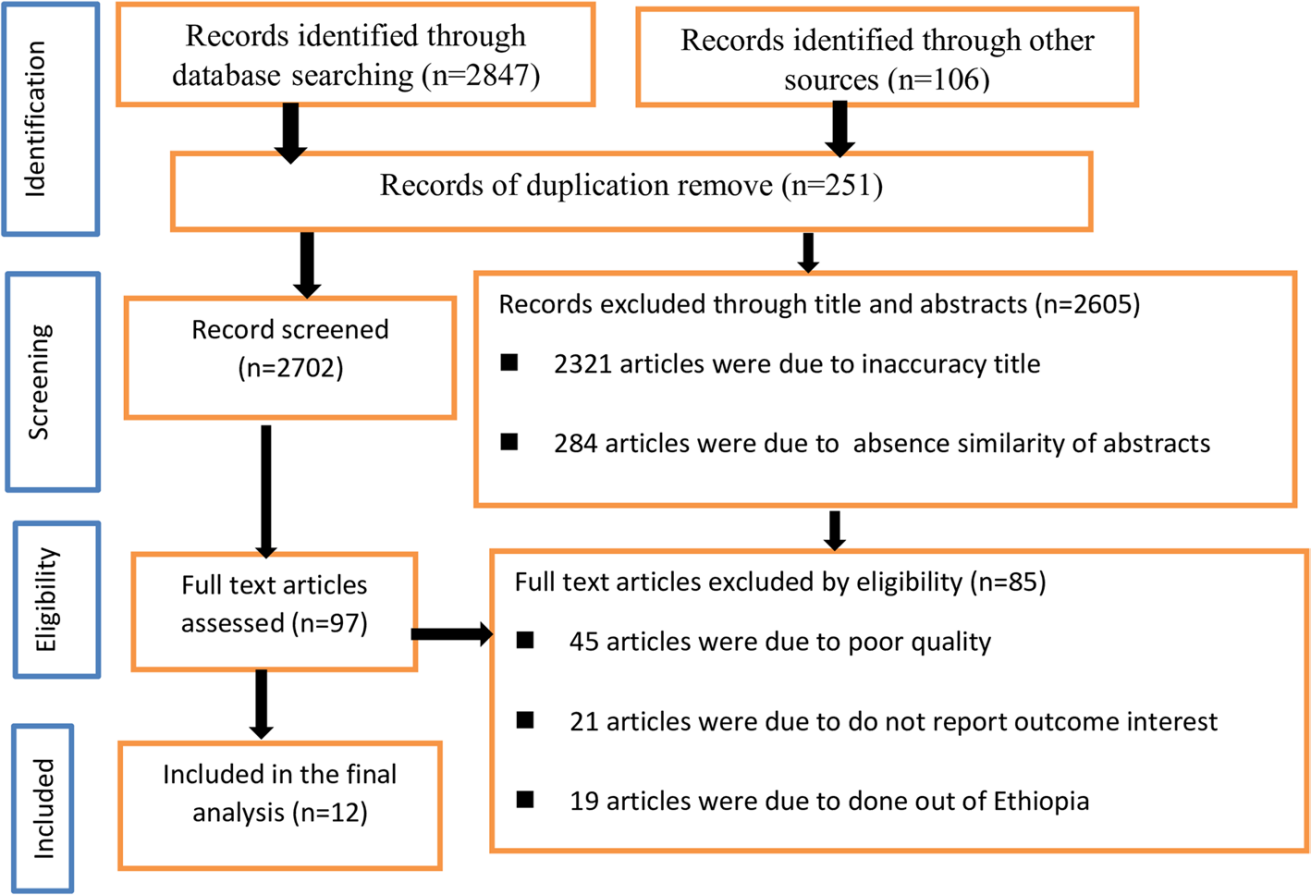
PRISMA diagram of selecting and including studies for a systematic review and meta-analysis for the prevalence of cardiovascular disease among diabetic patients in Ethiopia, 2021

In this review, 2847 articles were retrieved through internet searching (Pub Med, Google scholar, UCSF, and Ethiopian University repository online). One hundred six were identified through other sources. Totally, 2,953 articles were retrieved. Out of these, 251 duplicate records were removed from the review. Of the total articles, 2,321were due to inaccuracy title and 284 articles were due to absence similarity of abstracts were excluded from the review. After a full review of the articles, 74 were excluded by eligibility criteria. Finally, 12 articles that fulfill the inclusion criteria were used to determine the pooled prevalence of cardiovascular diseases and associated factors among diabetic patients in Ethiopia (Fig. 1).

### Characteristics of searched studies

This systematic review and meta-analysis included 3,784 study participants from 12 studies that evaluated the prevalence of cardiovascular diseases and associated factors among diabetic patients in Ethiopia. According to the regional distribution of the articles found through the search, five from Amhara, three from Oromia, and one each from SNNPR and Addis Ababa were included (Table 2).

**Table 2:** Characteristics of studies in a systematic review and meta-analysis on the prevalence of cardiovascular diseases and associated factors among diabetic patients in Ethiopia, 2022

Authors	Year	Region	study area	Study period	SD	SS	cases	PP (%)
Gebisto R. et al. [29]	2021	Oromia	JUMC	Sept 5, 2012-Feb 25, 2020	RS	424	65	15.3
Adane T., etal [30]	2020	SNNPR	Dilla RH	April to May 2019	CS	216	54	25.2
Tekabe A.,et al. [31]	2019	Harar	Hospitals	Feb-Mar 2017	CS	377	342	90.6
Senbeta G., et al. [32]	2020	AA	TASH	Nov 2015-Nov 2017	CS	306	196	64.1
Lemma D.,et al. [33]	2021	Harar	Hospital	Mar-APR 2018	RS	454	193	42.5
Hailemaryam A., et al [34]	2018	Amhara	UOGRTH	Apr 2-July 31, 2018	CS	272	47	17.3
Yonas A., et al. [35]	2020	Amhara	DTGH	Feb 1-Aug 30, 2019	CS	280	86	30.7
Birhanu W.,et al. [36]	2020	Amhara	UOGRTH	Feb-March 2018	CS	384	121	31.5
Solomon A.,et al. [11]	2019	Amhara	NORTH	Jan and Feb 2013	CS	231	82	35.3
Dessie A.,et al. [12]	2016	Oromia	JUTH	Feb 14-Apr 9 2014	CS	277	71	25.7
Solomon A.,et al. [37]	2018	Amhara	DRH	Jan 1-Apr 30, 2017	CS	256S	106	41.3
Daba A.,et al. [39]	2021	Oromia	AMCH	Mar 1–30, 2020	CS	307	84	27.2

*NB PP* Prevalence percentage, *SD* Study design, *SS* Sample size

### Heterogeneity and publication bias

The funnel plot test, I^2^, and its accompanying *p*-value were used to determine whether heterogeneity existed. The heterogeneity test was categorized as having low, medium, and high heterogeneity using values of 25%, 50%, and 75%. A random effect model of analysis was utilized for data with statistically significant heterogeneity. The statistical significance of publication bias was evaluated using Egger regression and the asymmetry test [29].

### Prevalence of cardiovascular diseases among diabetic patients (Systemic review and meta-analysis)

**Fig. 2:**
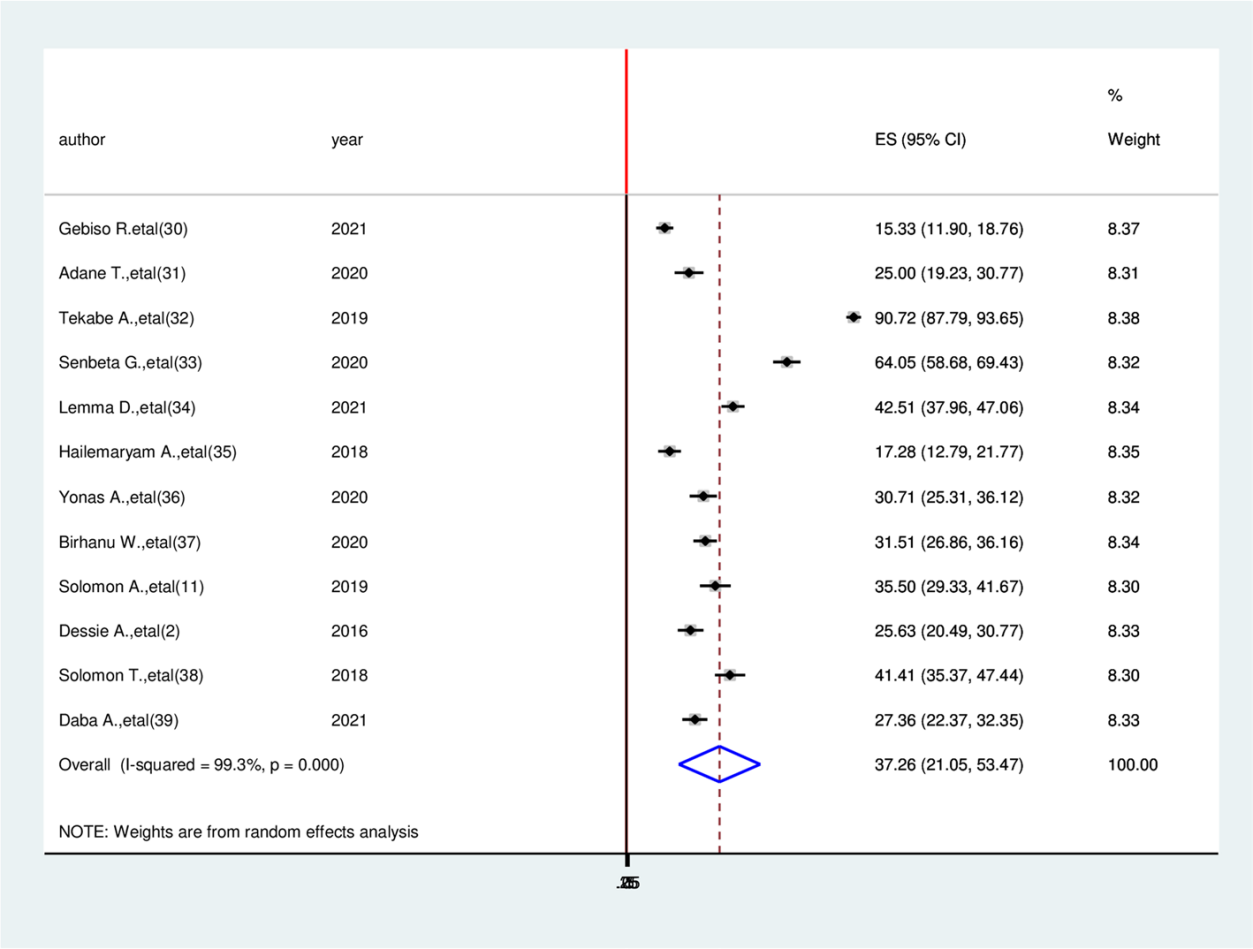
Forest plot of systematic review and meta-analysis on the prevalence of cardiovascular diseases and associated factor among diabetic patient in Ethiopia, 2022

The estimated pooled prevalence cardiovascular diseases among diabetic patients in Ethiopia was 37.26% (95% CI: 21.05, 53.47, I^2^ = 99.3%, *P* ≤ 0.001) (Fig. 2).

### Subgroup analysis of cardiovascular diseases among diabetic patients in Ethiopia

**Fig. 3:**
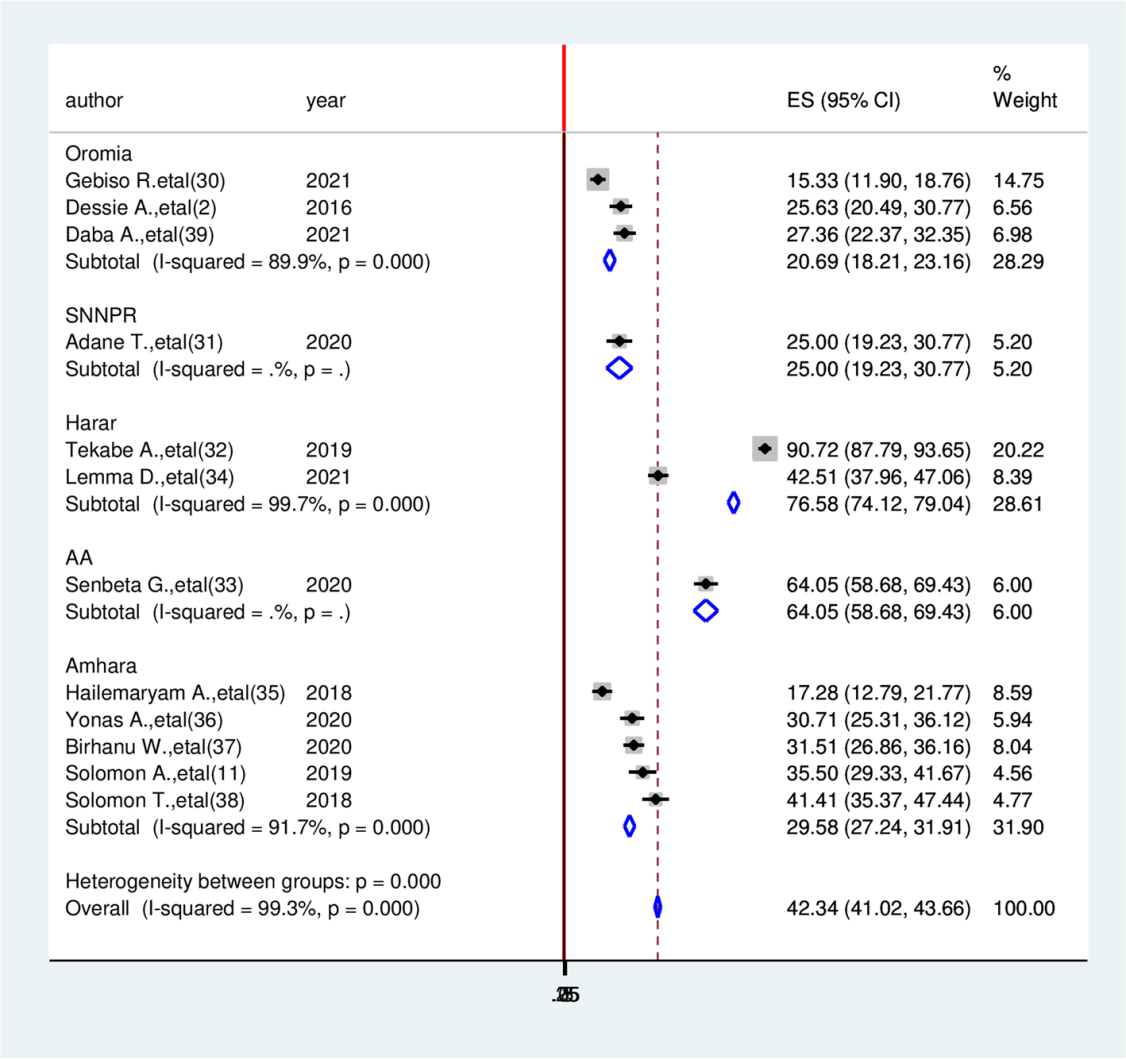
Subgroup analysis of systematic review and meta-analysis on the prevalence of cardiovascular diseases and associated factors among diabetic patients in Ethiopia, 2022

We have also performed subgroup analysis based on regions where the studies were carried out. The highest cardiovascular diseases were observed in Addis Ababa with a prevalence of 64.05 (95% CI: 58.68, 69.43) followed by Amhara region 29.58(27.24, 31.91) respectively. The least prevalent was observed in the Oromia region 20.69% (95% CI: 18.21, 23.16, I^2^ = 99.3%, *P* ≤ 0.001) (Fig. 3).

### Heterogeneity and publication bias

**Fig. 4:**
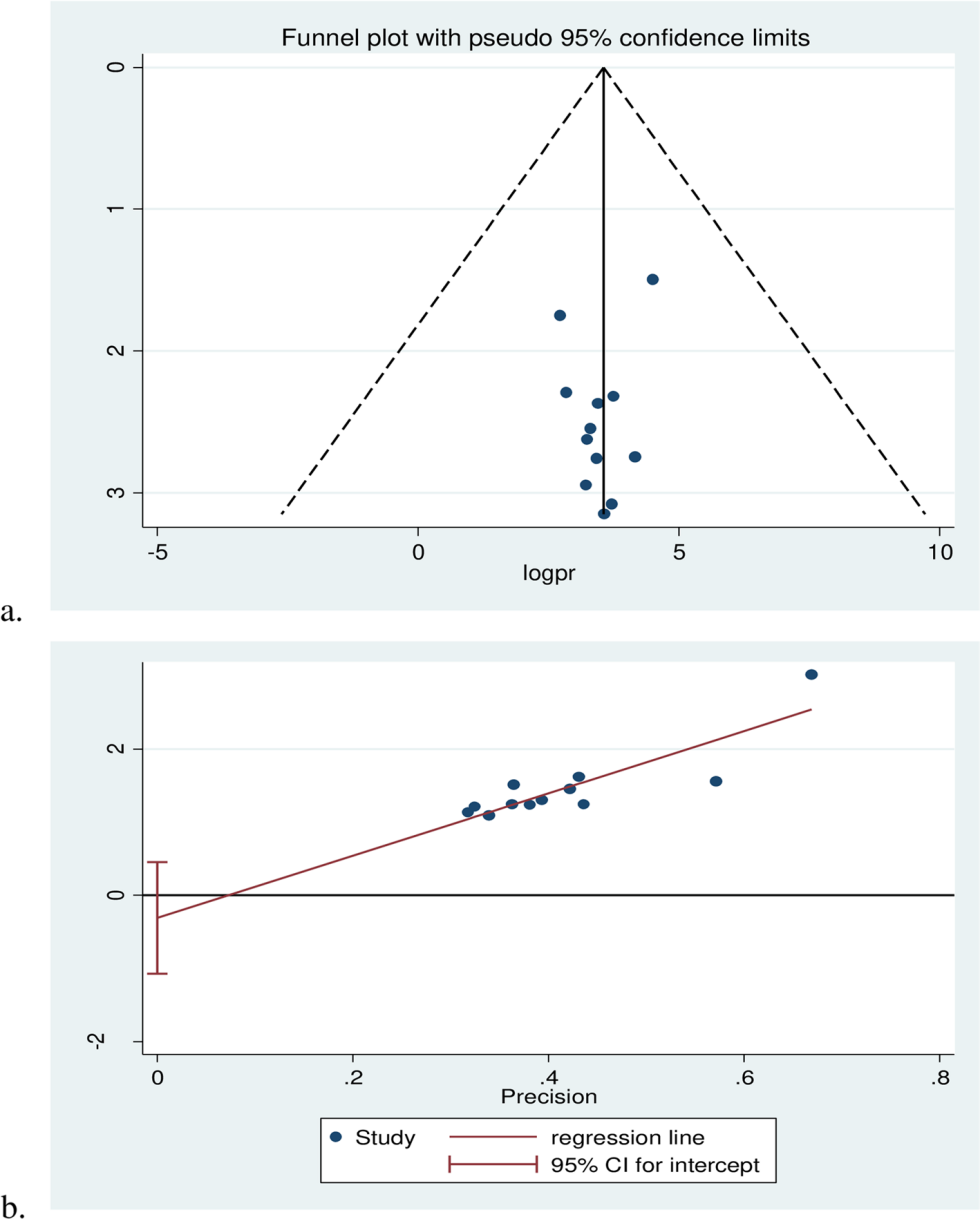
Funnel plot test (**a**) and egger test (**b**) of systematic review and meta-analysis on the prevalence of cardiovascular diseases and associated factors among diabetic patient in Ethiopia, 2022

The I^2^ (variation in ES attributable to heterogeneity) test results revealed that there was considerable heterogeneity with I^2^ = 99.3%, at *p*-value ≤ 0.001. The funnel plot results revealed that a systematic distribution of the included studies through inspection, which implied there was no potential publication bias and (Egger’s test: b = (- 0.90), *p* = 0.388) (Fig. 4).

### Sensitive analysis of cardiovascular diseases among diabetic patients in Ethiopia

**Fig. 5:**
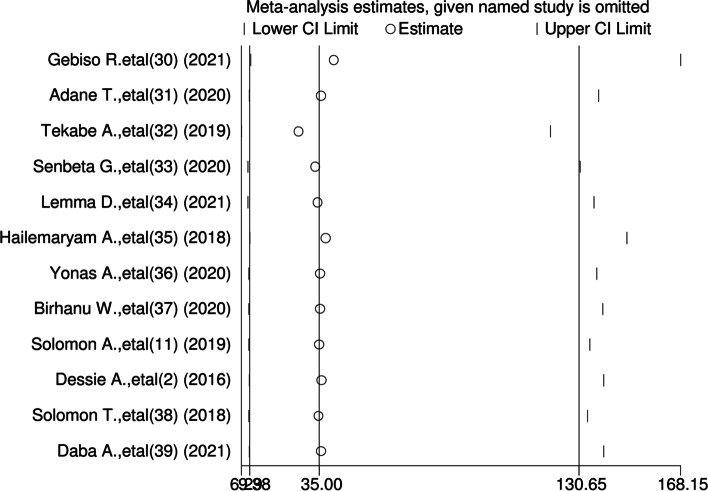
Sensitive analysis of a systematic review and meta-analysis on the prevalence of cardiovascular diseases and associated factors among diabetic patients in Ethiopia, 2022

We performed the test using a random effect, and the results showed that no single study influenced the overall pooled prevalence of cardiovascular diseases in Ethiopia (Fig. 5).

### Factors associated with cardiovascular diseases among diabetic patients in Ethiopia

By utilizing twelve pertinent studies, we have thoroughly reviewed and meta-analyzed the associated factors of cardiovascular diseases among diabetic patients in Ethiopia [2, 11, 31–38, 40, 41]. The study conducted in Ethiopia identified the contributing factors, which included the participants' age older than 60, body mass index > 24.9 kg/m^2^, the result of triglyceride (TG) ≥ 200 mg/dl, the status of Hypertension and the duration of DM condition were significantly and positively associated with cardiovascular disease.

**Fig. 6:**
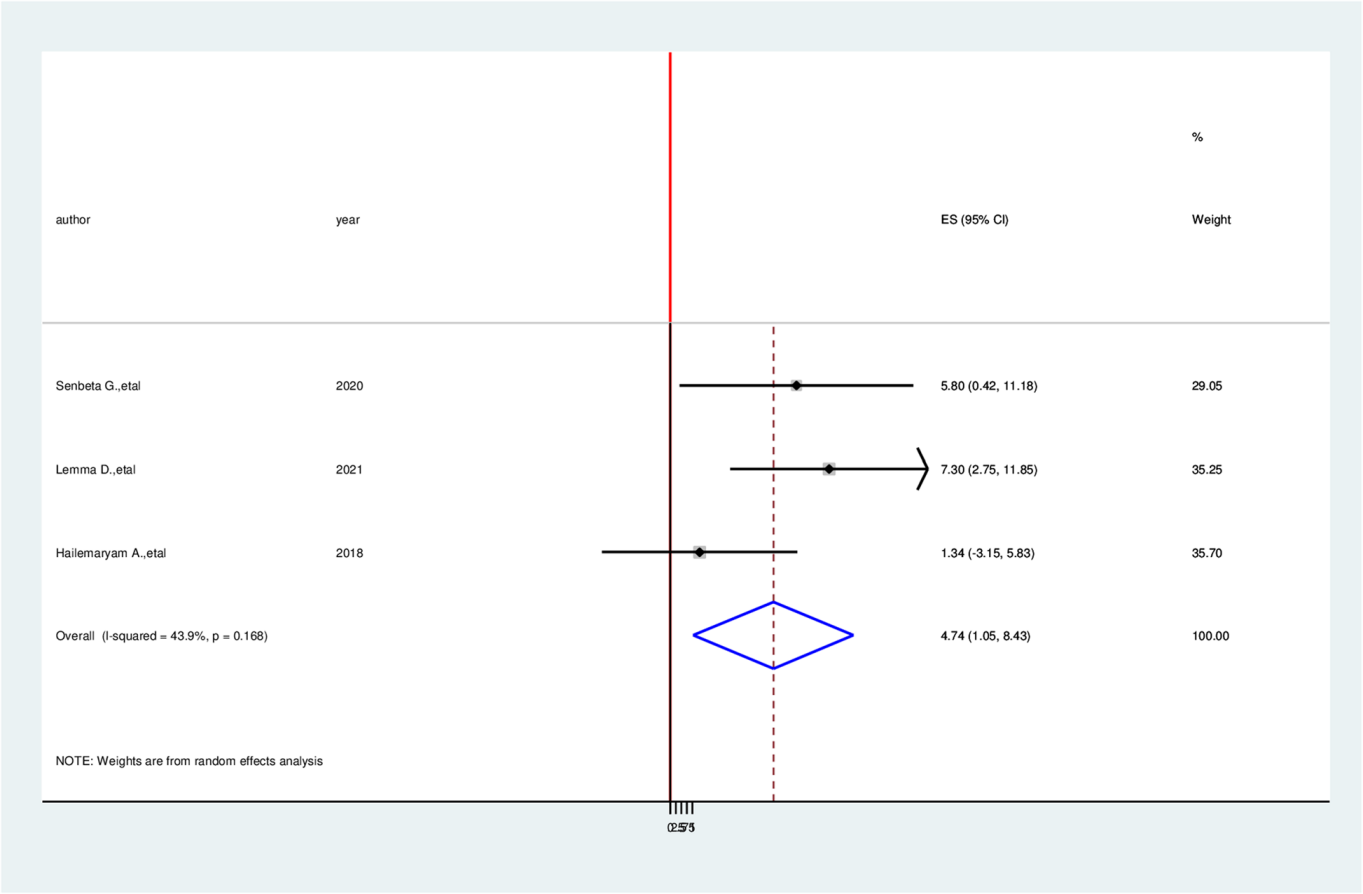
Forest plot on the association between age older than 60 years and cardiovascular disease among diabetic patients in Ethiopia, 2022

**Fig. 7:**
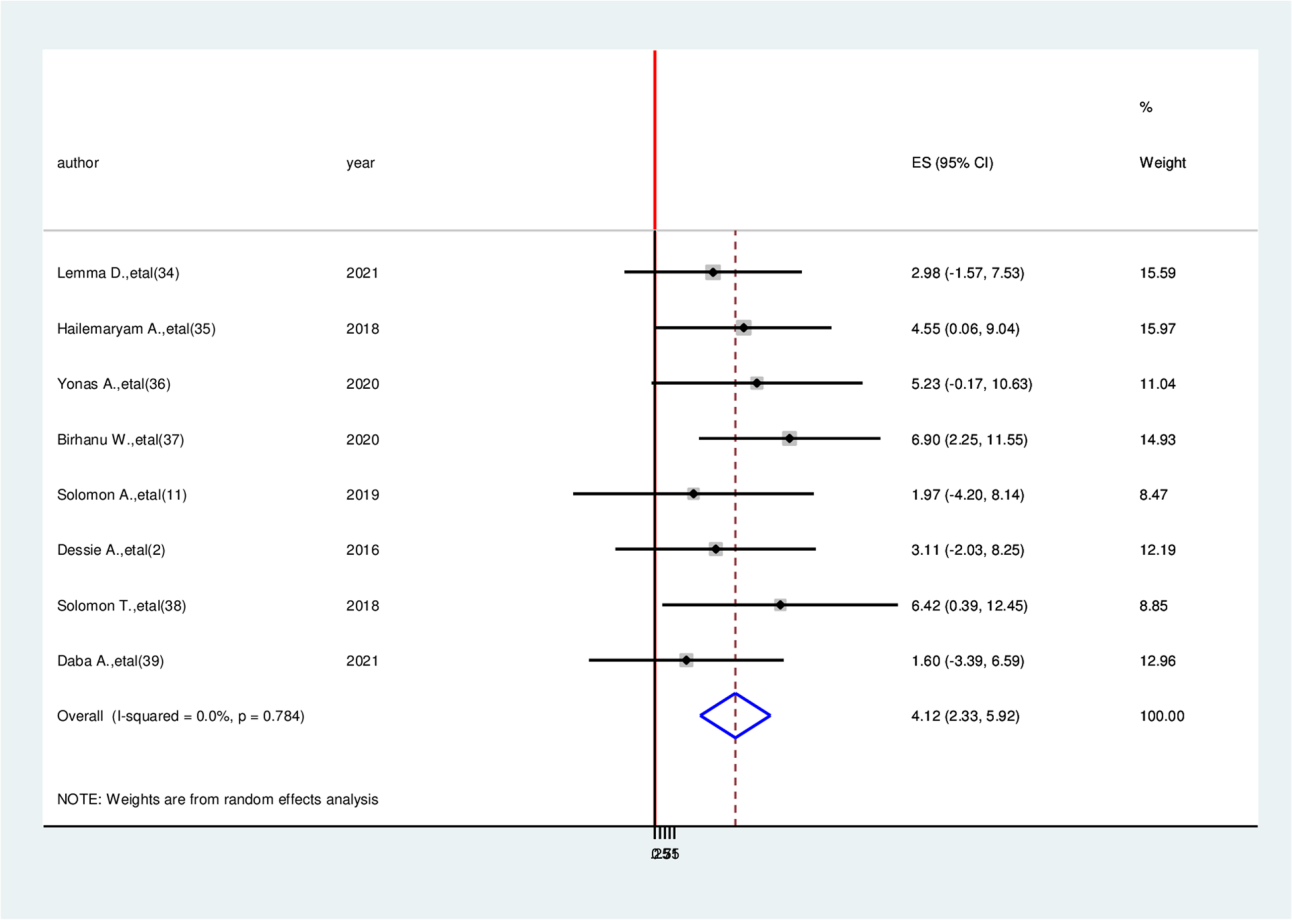
Forest plot on the association between higher body mass index and cardiovascular disease among diabetic patients in Ethiopia, 2022

According to this meta-analysis, patients with age older than 60 years were having 4.74 times higher chance of experiencing cardiovascular disease as compared with age less than this age category (AOR = 4.74,95%CI:1.05,8.43) (Fig. 6). Study participants who have body mass index more than > 24.9 kg/m^2^ were 4.12 times more likely to develop cardiovascular diseases than a patient with a normal body mass index was (AOR = 4.12, 95% CI: 2.33, 5.92) (Fig. 7).

**Fig. 8:**
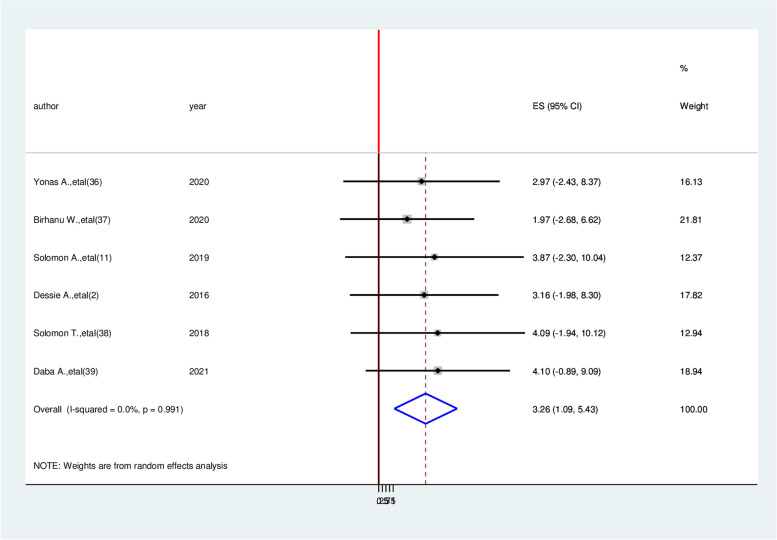
Forest plot on the association between hypertension and cardiovascular disease among diabetic patients in Ethiopia, 2022

**Fig. 9:**
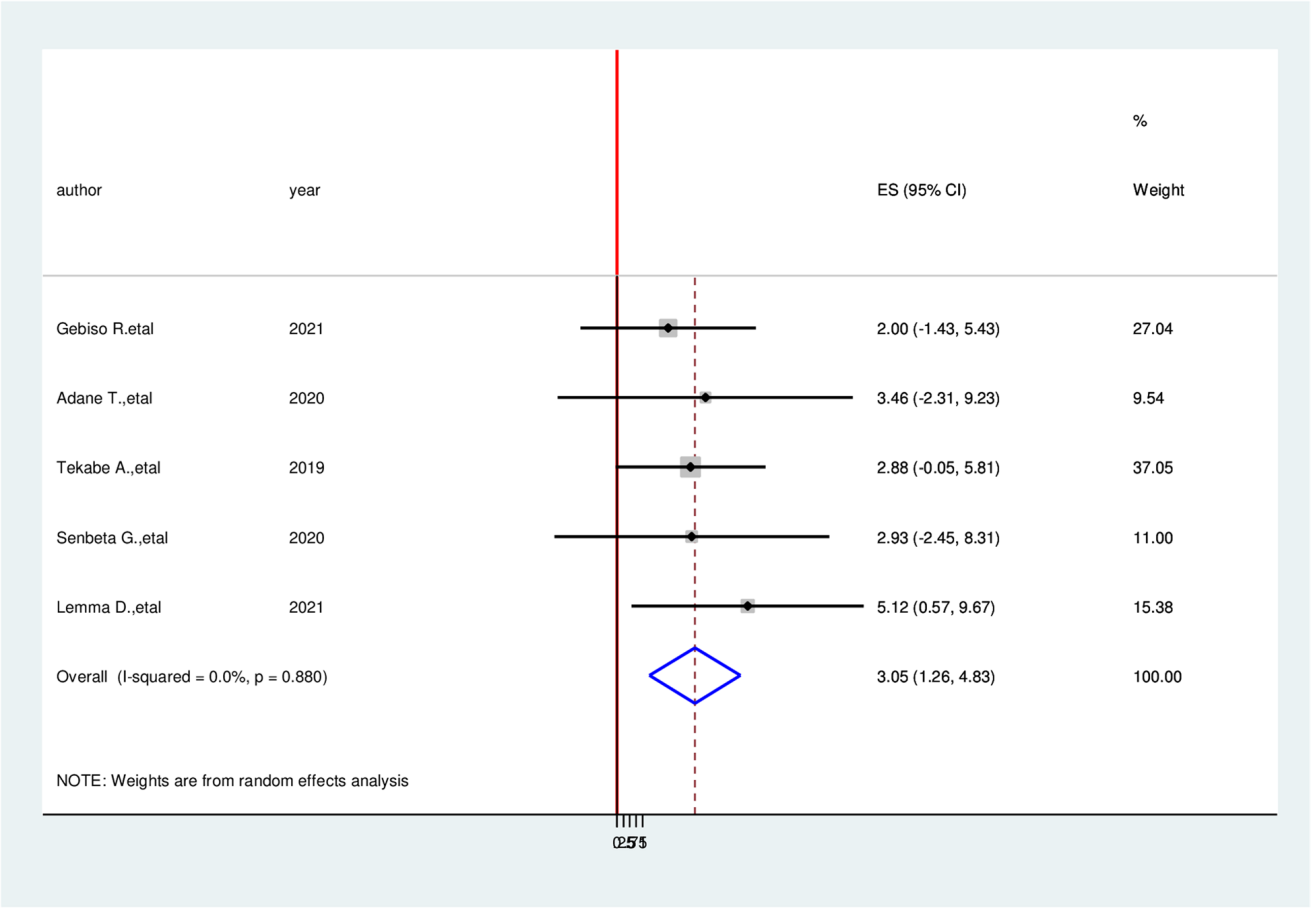
Forest plot on the association between triglyceride (TG) ≥ 200 mg/dl and cardiocascular disease among diabetic patients in Ethiopia, 2022

**Fig. 10:**
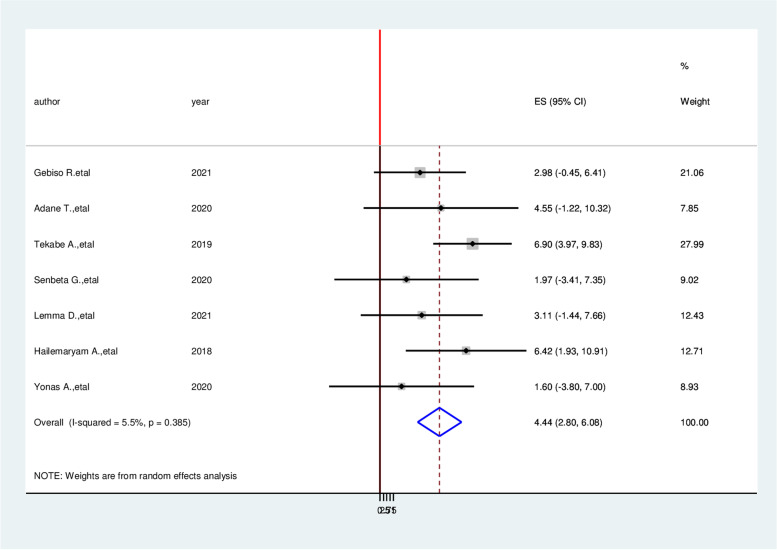
Forest plot on the association between duration of DM and cardiovascular disease among diabetic patients in Ethiopia, 2022

Patients who had a history of triglyceride > 200mg/dl had three times (AOR = 3.05, 95% CI: 1.26, 4.83) the likely hood of developing cardiovascular disease compared with diabetic patients who had normal triglyceride (Fig. 8). Similarly, the likelihood of acquiring cardiovascular disease among hypertensive patients is more than three times (AOR = 3.26, 95% CI: 1.09, 5.43) than non-hypertensive of diabetic patients (Fig. 9). On the other hand, Study participants who have more than four-year duration have 5.49 times more likely to cardiovascular diseases than a person who has less than four-year duration (AOR = 5.49, 95% CI:3.27,7.70) (Fig. 10).

## Discussion

Numerous researches have examined the prevalence of cardiovascular disease among Ethiopian diabetic individuals. The current systematic review and meta-analysis use twelve studies to determine the cumulative prevalence of the cardiovascular disease among diabetic individuals in Ethiopia. Cardiovascular disease was present in 37.26% of the population. The results of this review are consistent with a global systematic review and meta-analysis that was performed 32.2% [4], in an SNNPR research, 25.2% [31], Harar 42.5% [38], Region of Amhara 35.3% [11], and the region of Oromia 27.2% [34]. The reason could be due to duty similarity in study design, similarity in study area, similarity in sample methodology, and similarity in information sources.

This result exceeds that of a 15.3% study carried out in the Oromia region [32], and the Amhara region 17.3% [37].This difference can be the result of many study factors, such as diverse tools, multiple study designs, various tools, and different study participants.

The results of this review are less than those of the Harar research by 90.6% [41], In Addis Ababa 64.1% [40]. There are numerous potential defenses, including the study period, study location, changes in sample sizes, sampling procedures, and information sources.

In this meta-analysis, diagnoses made in patients older than 60 years of age were considerably more likely to have cardiovascular disorders than diagnoses made in patients younger than this age bracket. Previous studies provided evidence in favor of this study. As a person gets older, their blood vessels and heart may change, increasing their chance of developing. In addition, atherosclerosis and arteriosclerosis are more common when diabetes develops in older age [38]. Participants in the study who have been involved for longer than four years are much more likely to have cardiovascular problems than those who have been involved for less time. Participants in the study who had a body mass index more than or equal to 24.9 kg/m^2^ had a 4.12-fold increased risk of cardiovascular disease compared to those who had a normal BMI. This review has support by previous study [4]. In comparison to diabetic patients with normal triglycerides, patients with a history of triglycerides > 200 mg/dl had a threefold increased risk of developing cardiovascular disease. This conclusion was supported by earlier research. The source of this increase in triglycerides and LDL-C levels with aging may be owing to poor plasma clearance caused by decreased hepatic LDL-C receptor expression. While the precise mechanism of how aging affects blood lipid concentration is not entirely understood, it may be linked to the aging process and increased insulin resistance [42]. Similar to diabetic people, hypertension patients have a more than three-fold increased risk of developing cardiovascular disease. A significant contributor to mortality among those with T2DM, CVD accounted for over half of all fatalities during the research period. Stroke and coronary artery disease were the main causes. This is agreed with a research done before [4, 43]. On the other hand, study participants who have been involved for more than four years duration of DM are 5.49 times more likely to have cardiovascular problems than those who have been involved for less than four years. This review in lined with a thorough systematic review and meta-analysis that included data from 64 cohorts and more than 12 000 strokes found that the overall adjusted RR for total stroke related with DM was 2.28 (95% CI: 1.93–2.69) for women and 1.83 (95% CI: 1.60–2.08) for men [44].

### Strengths of this review

The use of numerous databases to search articles (both manually and electronically) and the uniform abstraction of material using a predetermined structure that helped to minimize mistakes were the review's strong points. Additionally, research from several Ethiopian regions was included in this meta-analysis. duration.

## Limitations of this review

Because the search was conducted only in English, bias may have existed. Primary studies may not be available in all parts of Ethiopia, making it challenging to draw conclusions about all of them.

## Conclusions

Diabetes patients are significantly more at risk for cardiovascular disease than the general public. Age above 60, BMI greater than 24.9 kg/m^2^, triglycerides greater than 200 mg/dl, hypertension, and diabetes duration longer than four years were identified in this research as independent predictors of cardiovascular disease in diabetic individuals. The findings highlight the need for a prospective study design with a longer follow-up period to evaluate the long-term effects of CVD predictors in diabetic patients. They also highlight the importance of paying attention to cardiovascular disease in diabetic patients with comorbidity like age over 60, BMI > 24.9kg/m^2^, triglyceride > 200 mg/dl, hypertension, and duration of DM > 4 years.

### Supplementary Information

**Additional file 1. **

## Data Availability

The data set supporting the conclusion of this article are included in the article.

## Abbreviations

AHA: American Heart Association
BMI: Body Mass index
CVD: Cardiovascular Disease
BP: Blood Pressure
T2DM: Type 2 Diabetes Mellitus
HTN: Hypertension
Kg/m^2^: Kilogram per square meters
LDL-C: Low- Density Lipoprotein cholesterol
NCDs: Non-communicable Diseases
TG: Triglyceride
WHO: World Health Organization
SNNPR: Southern Nation, Nationalities and Peoples’ of Region
